# Rotating shift work and menstrual characteristics in a cohort of Chinese nurses

**DOI:** 10.1186/s12905-016-0301-y

**Published:** 2016-05-04

**Authors:** Yizi Wang, Fang Gu, Mingfen Deng, Lan Guo, Ciyong Lu, Canquan Zhou, Shouzhen Chen, Yanwen Xu

**Affiliations:** Reproductive Center, the 6th Building, the First Affiliated Hospital of Sun Yat-sen University, Zhongshan 2 Road No. 58, YueXiu District, Guangzhou, 510080 Guangdong Province China; Department of Biostatistics and Epidemiology, School of Public Health, Sun Yat-Sen University, Guangzhou, Guangdong Province China; Nursing Department, the First Affiliated Hospital of Sun Yat-sen University, Zhongshan 2 Road No.58, YueXiu District, Guangzhou, Guangdong Province 510080 China

**Keywords:** Nurses, Shift work, Menstrual cycle, Circadian rhythm

## Abstract

**Background:**

Shift work disrupts the circadian rhythm and may cause menstruation disorders. This study assessed the impact of shift work on menstrual cycle in a population of Chinese nurses.

**Methods:**

Questionnaires on menstrual characteristics and shift schedules were sent to female nurses of the First Affiliated Hospital of Sun Yat-sen University (FAHSYSU) and Guanghua Hospital of Stomatology (GHHS), affiliated to Sun Yat-sen University. Part I was a cross-sectional study and included 139 nurses in GHHS who had regular 8:00–17:30 working (non-shift group), and 334 nurses from FAHSYSU who worked shifts, a response rate of 67.5 % and 59.6 %, respectively (age ≤ 50 years). Menstrual patterns were compared and age-adjusted relative risks of shift work were analyzed. Part II was a nested case–control study. Cases were nurses in Part I who had regular cycle with mean cycle length (MCL) of 25–31 days and but at least 3 days variation in MCL after starting shift work (*n* = 45). Controls consisted of 67 nurses with matching shift patterns and age, but no MCL changes. A control non-shift age-matched group consisted of 30 GHHS nurses with no MCL changes. A follow-up second questionnaire was sent 2 years later.

**Results:**

In Part I, the shift group had a significantly higher proportion of nurses with menstrual cycle irregularity. The proportion of nurses with a cycle of 25–31 days decreased from 81.7 to 67.8 % after changing to shift work. Logistic regression analysis showed that night shift frequency was the only risk factor associated with cycle shortening. After adjusting for age, MCL was shorter when night work was performed > 7 times per month. In Part II, the mean change in MCL in the case group, including prolongation or shortening, was 4.115 ± 2.084 days after shift working. In the 2 years’ follow-up, the MCL of the study group did not recover to the original length.

**Conclusions:**

Rotating shift work can increase the prevalence of menstrual cycle irregularity. Night shift frequency was the only risk factor associated with cycle reduced. Changes in MCL did not show recovery over a follow-up period of 2 years.

**Electronic supplementary material:**

The online version of this article (doi:10.1186/s12905-016-0301-y) contains supplementary material, which is available to authorized users.

## Background

The International Labor Organization has estimated that approximately 15–30 % of the workforce in developing countries comprise shift workers [1], and in the United States, about 15.2 million Americans are engaged in shift work [2, 3]. Disruption of the circadian rhythm in shift workers may affect follicular development and hormone secretion and disrupt the luteal phase, thus altering the menstrual cycle [4, 5]. In addition, shift conditions are also linked with a higher incidence of spontaneous abortion, spontaneous membrane rupture, pre-term birth, and reduced breastfeeding success [6]. Apart from the negative impact on the reproductive system, higher cancer risk, sleep/mood disorders, gastric disturbances, muscle aches, respiratory infections, and peptic ulcers have also been associated with shiftwork [2, 4].

Many nurses exposed to shift work are of reproductive age. In a study by Lawson et al.[3], rotating shift work and menstrual cycle patterns were investigated in 71,077 nurses aged 28–45 years. The relative risk of an irregular pattern increased slightly with increasing months of rotating shift work, with a 13 % increase in risk for every 12 months of rotating shift work. Asian nurses were more likely to have irregular cycles than white participants. In a subanalysis of nulliparous nurses with more than 20 months rotating shift work in 1993, the relative risk (RR) of an irregular cycle, short cycle length, and long cycle length was 1.21, 1.26, and 1.59, respectively.

Data from the Ministry of Health in China showed that there were 2.24 million registered nurses in 2012. With the increase in economic development, the work load of Chinese nurses in mainland China is increasing. However, few studies on the reproductive health of Chinese nurses have been carried out. The present study aimed to determine the impact of shiftwork on menstrual cycle patterns, and what factors were associated with cycle shortening in a cohort of nurses in China.

## Methods

### Study design and data collection

This study was conducted in two affiliated hospitals of Sun Yat-sen University, the First Affiliated Hospital of Sun Yat-sen University (FAHSYSU) and Guanghua Hospital of Stomatology (GHHS). Both hospitals were located in the same area of the same city. The FAHSYSU is a general hospital, containing various inpatient departments, and nurses have to work under shift schedules. The GHHS is a dentistry-specialized hospital without inpatient wards, and nurses work regular hours, 8:00–17:30, with no night or evening shifts.

This study comprised two parts. Part I was a cross-sectional study. Questionnaires mainly relating to menstrual characteristics and shift schedules were distributed to all registered female numbers (FAHSYSU, *n* = 939; GHHS, *n* = 308) in the two hospitals and collected by the head nurse in each department from June to December 2012. A total of 768 nurses returned the questionnaires, yielding a response rate of 61.6 %. The following participants were excluded: female nurses > 50 years of age (*n* = 6), nurses using hormonal pills or intrauterine devices as contraceptives (*n* = 128), nurses who had gone through menopause and/or were experiencing perimenopausal symptoms (*n* = 22), pregnant or breastfeeding nurses (*n* = 33), nurses who reported a diagnosis of menstrual dysfunction (e.g., Asherman’s syndrome, history of polycystic ovary syndrome, progesterone deficiency, endometriosis, hyperprolactinemia, and thyroid disorders) (*n* = 92), and nurses who had undergone hysterectomy and/or ovariectomy (*n* = 2). Because of the low number of respondents (*n* = 12) who had fixed evening or night shifts or other schedules, these two categories of shifts were omitted from the analysis. Finally, 334 nurses in the FAHSYSU and 139 in GHHS, with rotating shift patterns or day working only, were included in the study.

Part II was a nested case–control study. Cases included nurses from Part I whose mean cycle length (MCL) before shift working was within 25 to 31 days, and presented with a regular cycle but with at least 3 days variation in MCL after changing to shift work (*n* = 48). Two of these 48 nurses were not followed up, because of a change of jobs, and another with an extremely irregular menstrual cycle after shift work was excluded. The remaining 45 nurses completed a questionnaire after a further 2-years’ follow-up. A control shift group consisted of 67 nurses from the FAHSYSU who were matched for shift schedules and age with the cases, but had no MCL changes after shift work. A control non-shift group consisted of 30 age-matched nurses from GHHS who had no MCL changes after working. A second questionnaire was also sent to the control groups 2 years’ later.

### Questionnaire

The questionnaire contained items on age, height and body mass index (BMI), work duration, education level, sleep length on off days (hours), job satisfaction (which was determined through self-perception score (from 1 to 10) in this study and was classified in three group of low (score 1–4), medium (score 5–7), high (score 8–10) for job satisfaction), smoking (yes/no), drinking habit (categories; seldom/occasionally, monthly, weekly, two to three times per week, and daily), self-aware discomfort, including insomnia, dreaminess, fatigue, dizziness, memory loss, irascibility, palpitation, shortage of breath, chest distress, poor appetite or dyspepsia, and acne or hirsutism (yes/no), Self-rating Depression Scale (SDS) scores [7], shift work schedules (permanent daytime shift, fixed evening shift, fixed night shift, two-shift schedule, three-shift schedule, other schedule including night work, other schedule including evening work), age at first menstruation, pregnancy (yes/no), and menstrual cycle characteristics (including cycle duration, menstruation duration, and amount of bleeding, dysmenorrhea) (see Additional file 1).

In this study, the menstrual cycle pattern was obtained based on the question “How many days does it usually take from the first day of your menstruation in a cycle to the first day of menstruation in the next cycle?” The onset of menstrual cycle was defined as the first of 2 consecutive days with onset of bleeding, where bleeding was more than spotting on at least one day [8]. This variable was divided into three categories during data processing: < 25 days, 25–31 days, and > 31 days [3, 9]. Having > 7 days for changes in the menstrual cycle duration was defined as irregular [10].

Changes in MCL were determined from the question “Normally, whether the number of days from the beginning of a cycle to the next cycle is as expected (identical) or not? If menstruation occurred earlier or later than expected, what was the difference in days?” We categorized answers as follows: extremely regular (no more than 1–2 days’ difference from expected), very regular (maximum of 3–4 days’ difference), regular (within 5–7 days) [3].

The questions “How many days does menstrual bleeding take on average?” and “What is your total menstrual quantity in one cycle on average?” were asked to determine bleeding duration and amount (less than 10 mL defined as hypomenorrhea, 10–80 mL or 1–6 tablespoons of menstrual fluid defined as moderate, and > 80 mL defined as heavy) [11].

Irregular bleeding was defined as bleeding or spotting between cycles (intermenstrual bleeding or spotting) (yes/no) [12]. Dysmenorrhea was defined as at least 2 days of low back pains or/and abdominal discomforts during menstrual bleeding [10] and was categorized as three options: mild (no effect on normal work and life), moderate (slight effect on normal work but no need for medicines) or heavy (marked effect on normal work and life or have to use of non-steroidal anti-Inflammatory drugs to alleviate pain).

The question “Have you noticed differences in menstrual characteristics between before and after starting work?” was asked to determine whether menstrual characteristics were affected by work schedules. If the answer was yes, the following were determined: a) Irregular menstrual cycles prior to working (yes/no); b) changes in menstrual cycle length and menstrual bleeding duration after starting work; c) changes in amount of flow after starting work (categories: no change, less or more); and d) dysmenorrhea intensity (no change, alleviate, or aggrevate).

The questions “How many years of work included evening and/or night shifts during your whole life?” and “How many evenings did you work each month on average during the past 12 months?” were asked. The frequency of night work was divided into six categories: no less than 7 nights, or 6 nights, or 5 nights, or 4 nights, no more than 3 nights, and none per month. The frequency of evening work was determined in the same manner.

Shift schedules were defined as all types of shifts, including fixed evening shifts, fixed night shifts, and rotating shifts but did not include fixed daytime shifts [13]. Fixed evening shifts were worked from 4:00 p.m. to 12:00 a.m., and fixed night shifts from 12:00 a.m. to 8:00 a.m. Rotation shifts referred to the following two types. Type 1 was a two-shift rotation (lasting 12 h per shift), including an evening schedule (evening shift from 12:00 p.m. to 12:00 a.m.) and night schedule (night shift from 12:00 a.m. to 12:00 p.m.). The other type was a three-shift rotation (lasting 8 h per shift, day shift from 8:00 a.m. to 4:00 p.m., evening shift from 4:00 p.m. to 12:00 a.m., and night shift from 12:00 a.m. to 8:00 a.m.). One or two off days were always followed with a shift rotation, as a rapid shift rotation. A 3-month schedule of rotating shifts was always alternated with only daytime work lasting at least 1 month, as a slow shift rotation. Based on the three questions regarding shift work, three measures of exposure to night and/or evening work were used: a) evening or night work now or earlier (yes/no); b) number of years of work periods including evening or night shifts in total in the past; c) type of shift schedules (multiple-choice question); d) number of evenings and nights worked per month during the past 12 months (six categories were used as mentioned above). In the present study, most nurses from FAHSYSU worked rotating shift schedules. Only four nurses worked fixed evening shifts, three nurses worked fixed evening shifts, and five worked under schedules different from those mentioned above. The nurses working fixed shift schedules, rather than rotating schedules, were all newly recruited (under shift schedules for less than 6 months, ranging from 3 to 5 months). These nurses were excluded from the analysis.

### Ethical considerations

Ethical approve was granted by the Faculty of Medical Research Service Ethics Committee, the First Affiliated Hospital of Sun Yat-sen University, China. All voluntary participants gave written informed consent to the use of job number with their data on investigation, and for publication of quotes from the interviews. Also, they could exit the study at any time they wanted. No compensation was offered for participation in this study.

### Statistical analysis

Quantitative variables were concluded to have a normal distribution according to the Kolmogorov–Smirnov test. The Student *t*-test was used to compare quantitative data. For qualitative variables, the differences between the shift and non-shift groups were determined using the chi-squared test, where *P* < 0.05 was considered to be statistically significant. Also, the logistic regression analysis was used to adjust the confounding factors (including BMI, age and perceived job satisfaction) and to more accurately assess the relationship between exposure to shift working and menstrual cycle length. Both the stepwise and entry methods were applied to filter risk factors; the collinearity test was applied to distinguish parallel impacts of shifts. The outcomes are represented by the odds ratio (OR) with 95 % confidence interval (CI). In Part II, the paired-sample *t*-test was applied to compare MCL among groups before shift working, after shift working, and after 2 years’ follow-up. Because the distribution of the fluctuation in MCL after 2-year follow-up was skewed, the Kruskal–Wallis H test was used. The fluctuations were classified into three grades (zero, < 2 days, and other) and regarded as ordinal categorical variables. All statistical calculations were two-sided and performed with SPSS version 20.0 (IBM Inc., Armonk, NY, USA).

## Results

In Part I of the study, sociodemographic and occupational categories in the shift and non-shift groups were compared (Table 1). Nurses in the shift group were older (*P* = 0.048) and had a higher BMI than those in the non-shift group (*P* = 0.003). There was a significant difference in educational attainment, with 79 % of nurses in the shift group having a bachelor degree while about half of nurses in the non-shift group had only completed junior college (*P* < 0.001). However, there were no significant differences between the groups with regard to weight fluctuation over 3 years, duration of work, mean sleep length on off-duty days, age of menarche, and history of pregnancy. Almost all nurses denied cigarette consumption, and very few were habitual drinkers after rotating shifts. The shift group expressed less satisfaction with their current life and felt less satisfied with the job (*P* < 0.001), in accord with significantly higher psycho-neural signs (*P* < 0.001).

**Table 1:** Comparison of sociodemographic and occupational characteristics between non-shift and shift nurses

Variables	Group		*P*-value
	Non-shift (*n* = 139)	Shift (*n* = 334)	
Age (years), mean (SD), range	28.08 (5.05), 21–45	29.49 (5.30), 21–46	0.048
BMI (kg/m^2^), mean (SD), range	19.73 (1.92), 15.4–27.3	20.49 (2.62), 15.2–33.5	0.003
Weight fluctuation (kg); mean (SD), range	2.72 (6.48), −5–15	1.64 (6.07), −10–20	0.176
Age at menarche (years); mean (SD), range	13.42 (1.38)	13.51 (1.23)	0.494
Work duration (years); mean (SD), range	6.82 (5.25), 1–26	7.68 (6.03),1–24	0.123
Sleep length (hours); mean (SD), range	7.32 (1.05), 6–11	6.78 (1.32), 5.5–11	0.942
Educational level; n (%)			
Technical secondary school degree	10 (7.2 %)	2(0.6 %)	<0.001
Junior college graduate degree	69 (49.6 %)	63 (18.9 %)	
Bachelor degree	6 0 (43.2 %)	264 (79.0 %)	
Master degree and above	0 (0 %)	5 (1.5 %)	
Perceived job satisfaction; n (%)			
Low	1 (0.7 %)	57 (17.1 %)	
Medium	18 (12.9 %)	135 (40.4 %)	<0.001
High	120 (86.3 %)	142 (42.5 %)	
Perceived discomfort signs; n (%)			
Insomnia, dreaminess, fatigue, dizziness, memory loss	78 (56.5)	287 (85.9)	
Irascibility, palpitation, shortage of breath, chest distress	39 (28.3)	203 (60.8)	<0.001
Poor appetite or dyspepsia	20 (14.5)	150 (44.9)	
Acne or hirsutism	21 (15.2)	126 (37.7)	
Pregnancy history; Yes (%)	29 (21.5)	84 (25.1)	0.319
Smoking; Yes (%)	0	0	
Alcohol consumption; Seldom (%)	330 (98.8)	129 (92.8)	0.383
Standard SDS score; mean (SD), range	32.0 (5.01), 25–50	37.5 (4.43), 30–52.50	0.024

*SD* standard deviation

Table 2 provides information on menstrual patterns before and after starting work (in the non-shift group) or starting work rotation (in the shift group). Although fewer nurses presented with dysmenorrhea before starting shift work compared with the non-shift group before working, dysmenorrhea occurred more frequently after shift working in the shift group (*P* = 0.01). The shift group also had a significantly higher proportion of nurses who had irregular menstrual cycles, and the proportion of nurses with a 25–31-day cycle decreased significantly from 81.7 to 67.8 %. Among 334 nurses in the shift group, 31 had a shortened regular cycle, while 17 had a longer cycle after starting shift work. There was a significant difference in the changes in MCL between the non-shift and shift groups (*P* = 0.028).

**Table 2:** Menstrual patterns of nurses in non-shift (*n* = 139) and shift (*n* = 334) groups

Before					After			
Variables	Group		*χ* ^*2*^	*P*-value	Group		*χ* ^*2*^	*P*-value
	Non-shift	Shift			Non-shift	Shift		
Dysmenorrhea	92 (66.2)	171 (51.2)	8.933	0.003	103 (74.1)	204 (61.1)	--	0.01^*^
Mild	53 (38.1)	82 (24.6)	4.651	0.098	61 (43.9)	22 (6.6)	81.458	0.000
Moderate	27 (19.4)	73 (21.9)			32 (23.0)	135 (40.4)		
Heavy	12 (8.6)	16 (4.8)			10 (7.2)	47 (14.1)		
Irregular menstrual cycle	13 (9.7)	52 (16.6)	3.562	0.059	20 (14.9)	78 (24.8)	5.403	0.020
Regular menstrual cycle								
≤ 24 days	10 (8.3)	10 (3.8)	4.470	0.107	14 (12.3)	26 (11.0)	7.119	0.028
25–31 days	99 (81.8)	214 (81.7)			89 (78.1)	160 (67.8)		
≥ 32 days	12 (9.9)	38 (14.5)			11 (9.6)	50 (21.2)		
Amount of flow								
Hypomenorrhea/reduction	12 (8.6)	42 (12.4)	4.686	0.096	10 (7.2)	42 (12.6)	17.376	0.000
Moderate/No changes	118 (84.9)	254 (76.0)			116 (83.5)	215 (64.4)		
Heavy/increase	9 (6.5)	38 (11.4)			13 (9.6)	77 (35.8)		
Bleeding during menstrual cycle	3 (2.2)	8 (2.4)	0.024	0.876	9 (6.5)	37 (11.1)	2.669	0.124

Variables are n (%)

*As a statistical difference had been found in dysmenorrhea between shift and non-shift groups, the dysmenorrhea rate was only compared in the shift group before and after the change to shift work

Logistic regression analysis showed that night shift frequency was the only risk factor associated with cycle shortening. After adjusting for age, MCL was shorter when night work was performed > 7 times per month (Table 3).

**Table 3:** Age-adjusted association between rotating shift work and menstrual cycle

Variables	Menstrual cycle length ≤ 24^c^	Menstrual cycle length ≥ 32^cb^
	*n* = 26	*n* = 48
	OR 95 % CI	OR 95 % CI
Shift work duration (months)		
0^a^	1.00	1.00
1–9	0.88 (0.72–0.91)	1.02 (0.93-1.05)
10–19	1.13 (0.84–1.62)	1.25 (0.94-1.31)
20+	0.97 (0.83–1.29)	1.08 (0.76-1.47)
Frequency of evening work/month		
0^a^	1.00	1.00
1–3	0.76 (0.54–1.06)	0.43 (0.35-0.56)
4–6	1.01 (0.92–1.47)	1.26 (0.83-1.57)
7+	0.92 (0.97–1.25)	1.04 (0.86-1.19)
Frequency of night work/month		
0^a^	1.00	1.00
1–3	0.76 (0.53–0.92)	0.49 (0.31-0.72)
4–6	1.48 (0.77–1.91)	1.44 (0.69-1.82)
7+	1.76 (1.32–2.28)	0.91 (0.85-0.98)
Age (years)		
21–30	0.34 (0.29–0.51)	1.27 (1.20-1.34)
31–35	0.60 (0.52–0.93)	1.05 (0.92-1.15)
36–40^a^	1.00	1.00
41–46	1.76 (1.02–2.04)	0.53 (0.49-0.61)
BMI (kg/m2)		
<18.5	1.22 (0.70-2.36)	1.79 (1.63-1.94)
18.5-24.9^a^	1.00	1.00
25-29.9	1.06 (0.81-1.74)	1.18 (1.06-1.26)
30-35	2.03 (0.75-3.97)	1.24 (1.09-1.43)
Perceived job satisfaction		
High	0.58 (0.30-0.79)	0.83 (0.67-0.87)
Medium^a^	1.00	1.00
Low	2.11 (0.94-3.69)	1.74 (0.94-1.82)

^a^ Reference category;

^b^ Exclusion of women (*n* = 2) with regular menstruation but cycle length longer than 45 days

^c^ Reference category presented by menstrual cycle length 25–31 days, *n* = 160

In Part II, the characteristics of 45 nurses (case group) from Part I with MCL of 25–31 days before starting shift work, and a regular cycle but with at least 3 days variation in MCL after shift working, are compared with those of shift and non-shift control groups, both not only with a regular cycle but MCL also remaining intact at the first survey (Table 4). Before starting shift work, the average MCL of the case group was 27.9 ± 1.27 days. After starting shift work, at the first survey, cycle length was reduced by 3.3 ± 1.89 days in 28 of the 45 nurses (62 %) and their mean MCL was shortened to 24.9 ± 2.23 days, while 17 (38 %) showed a prolongation of 5.3 ± 1.90 days leading to average MCL of 32.6 ± 2.30 days. Collectively, the mean change in MCL in the case group, including prolongation or shortening, was 4.115 ± 2.084 days. The number of nurses with MCL of 25–31 days was reduced to 18, and then to 14 after a further 2 years of rotating shift work. In addition, changes already detected at the first survey seemed to be irreversible, as the MCL of the nurses in case group did not rebound to the initial duration after another 2 years of rotating shift work. Statistical differences were only detected in the distributions of MCL before shift working compared with that at the first survey and after 2 years’ follow-up, while the distributions in the latter two showed with no statistical difference.

**Table 4:** Characteristics of subgroups after 2 years’ follow-up

Variables	Case group	Control group			*P*-value
	Shift (*n* = 45)	Shift controls (*n* = 67)	Non-shift controls (*n* = 30)		
Age (years); mean (SD), range, days	27.4 (3.26), 22–35	28.7 (2.53), 24–34	27.2 (2.86), 23–34	F = 3.60	0.050
BMI (kg/m^2^); mean (SD), range	19.5 (1.50), 16.8–23.8	20.3(1.75);16.41-23.94	19.7 (2.07), 15.94–24.88	F = 2.78	0.065
Duration of shift work (years); mean (SD), range	5.2 (2.99), 2.5–9.0	6.1 (1.78), 3–10.5	4.2 (1.67), 2.0–8.0	F = 13.3	0.000
Number of night shifts/month; mean (SD), range	3.2 (1.39), 1.0–6.0	3.8 (1.29), 1.5–7.0	––	t = 1.71	0.089
Number of evening shifts/month; mean (SD), range	4.5 (1.23), 2.5–7.5	3.7 (1.45), 1.5–8.0	––	t = 3.08	0.003
Sleep length (hours); mean (SD), range	6.32 (1.47), 5–11	6.18 (1.23), 5–10.5	7.01 (1.65), 5.5–10	F = 7.29	0.01
Standard SDS score; mean (SD), range	37.8 (5.31), 26.0–48.75	36.4 (4.55), 26.25–47.50	35.0 (5.15), 23.75–42.50	F = 3.02	0.052
Perceived job satisfaction; n (%)					
Low	5 (11.1 %)	8 (11.9 %)	0	*χ* ^*2*^ = 29.83	0.000
Medium	37 (82.2 %)	54 (80.6 %)	16 (53.3 %)		
High	3 (6.7 %)	5 (7.5 %)	14 (46.7 %)		

**Fig. 1:**
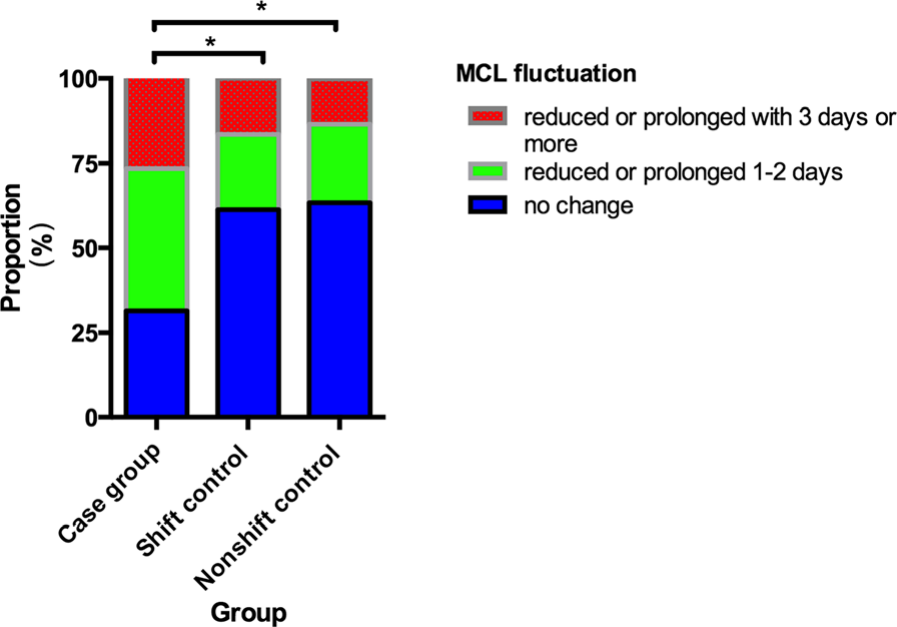
Histogram about further changes (reduced or prolonged) of MCL of case group (*n* = 45) and control groups (shift-control group, *n* = 67; nonshift-control group, *n* = 30) in Part II after another 2 years’ follow-up in a proportion form. Significant differences on distribution of MCL fluctuation existed between the case group and each control group. * Significant differences

The proportion concerning further changes (reduced or prolonged) of MCL over 2 years’ follow-up was displayed in Fig. 1. The absolute value (ABS) of fluctuation was classified into three grades defined as 0 (no change), 1–2 days (reduced or prolonged 1 to 2 days) and ≥3 days (reduced or prolonged 3 days or more). There were significant differences in the MCL fluctuation in the case group compared with each control group (controlled shift group: T = 3333.5 and *P* = 0.004; non-shift group: T = 913.0 and *P* = 0.008) over 2 years’ follow-up period. There was no statistically significant difference between the two control groups regarding MCL fluctuation (T = 1440, *P* = 0.787).

## Discussion

The menstrual cycle pattern is regarded as a pivotal indicator of reproductive health. It is mainly regulated by the hypothalamus-pituitary-ovary axis. Shift work, which may interrupt the normal function of the biological clock, is considered to be one of the factors contributing to the changes in the menstrual cycle. The present study provided further evidence to show that shift work has a negative impact on the normal regular cycle length, and that the change in cycle length does not recover to the original length after prolonged shift working over 2 years.

The exact mechanism to explain how daily circadian changes affect the regulation of the monthly menstrual cycle remains obscure. Recently, Archer et al. reported that sleep disturbance can reduce transcription of circadian-associated genes from 6.4 to 1.0 % [14]. Shift work may disturb the autonomous circadian oscillator of the gonadotropin-releasing hormone neurons, and pituitary and follicle cells, thus leading to a disturbance in the secretion of endocrine hormones [15]. Shift work affects the routine oscillatory rhythm and melatonin levels, which can influence the development of follicles by suppressing the exogenous secretion of estrogen [16–18]. A recent study detected significantly higher urinary prolactin levels, which could be associated with prolongation of the follicular phase, and even anovulatory bleeding in a shift group compared with non-shift workers [8, 9]. In addition, a surge in luteinizing hormone that is supposed to be limited to the hours of sleep and is correlated with cortisol levels may be affected by adverse sleep-waking patterns and shift-associated stress [6, 19]. Shift work may also make an impact on dietary habit and quality. Erratic meal patterns or skipping meals, increased consumption of energy later in the day and increased snacking were common, with multiple snacks being consumed during the night shift in place of a full meal were common. The significance of these altered dietary habits must be considered, since it has been proved by convincing evidence that night time eating can cause disruptions to endogenous circadian rhythms, compared with day time eating [20]. Changed eating habit may leave shift group more susceptible to developing metabolic syndrome (MetS) [21], which is particularly in relation to risk of insulin resistance and type 2 diabetes mellitus (T2DM) [22]. All referred disorders, ordinarily companied with noticed weight gainor loss (indirectly) [23], might give clues for explanation on cycle irregularity, cycle length shorting (eg. luteal phase defect)/prolongation (eg. retardation of follicular development) [24], or other manners of menstruation problems.

Although there are studies that found no association between night work and irregular menstruation or cycle length variation [25], a systematic review of 16 independent cohorts from 15 studies (a total of 123,403 women) affirmed that shift work could increase the menstrual disruption rate to 16.05 % from 13.05 % in non-shift workers [26].

The largest epidemiological study, which was carried out in 71,011 nurses in the United States, suggested that there was a dose response relationship in rotating shift work with cycles of 40 days or more, and reported a 25 % increase in the risk of changes in MCL for every 12 months of rotating shift work. A trend test also showed a 13 % increased risk of an irregular pattern for every 12 months of rotating shift work. Nurses with 20 or more months of rotating shift work were more likely to have irregular cycles (>7 days variability) (adjusted RR, 1.23 [95 % CI, 1.14–1.33]; they were also more likely to have a cycle length < 21 days (RR, 1.27; 95 % CI, 0.99–1.62) or > 40 days (RR, 1.49; 95 % CI, 1.19–1.87) (both compared with nurses with MCL 26–31 days) [3]. Race differences may exist, as Asian nurses were more likely to have irregular cycles and shorter cycles than white participants. However, the study was mainly focused on shift-working for less than 20 months, and shift frequency was not taken into account. The latter should be regarded as an important index to evaluate the load of shift work. Furthermore, the data on the Asian population were quite limited.

Only a few studies have been carried out in Asian nurse populations. One study reported that 53 % of women noted menstrual changes when working shiftwork, with MCL shortened in 3 %, lengthened in 9 %, and variable in 10 % [2]. It was not a detailed investigation but did suggest that changes in menstrual function may be a marker of shift work intolerance. In another study, a higher prevalence of irregular ovarian cycle patterns (40 %) and changes in the regular monophasic ovarian cycle (42 %) were demonstrated in nurses who worked rotating shifts, but only 50 nurses working under rotating shift schedules participated in that study [27]. Clear definitions of irregularity and rotation cycle were not mentioned, so the study cannot be adequately compared with the present one.

In 2005, Chung et al. studied 200 nurses from five different facilities (including wards, emergency rooms (ERs), and intensive care units (ICUs)) in Taiwan where the shift schedule was identical to the three-shift rotation in our study, and found that nurses working rotation shifts showed a trend for a reduction in MCL to < 25 days. The proportions of nurses with an MCL < 25 days in wards, ERs, and ICUs, were 23.1 %, 25 %, and 22.2 %, respectively, and were double that of our study. Additionally, it was also found that 45.2 % of nurses in the three units reported irregular menstrual cycles, while the proportion in our study was 24.8 %. In the previous study, the highest prevalence of MCL of < 25 days and of irregular menstrual cycles were 60 % and 54.5 %, respectively and occurred in nurses working permanent night shifts (12:00 a.m. to 8:00 a.m.), followed by nurses under rotating shift schedules [9]. This was in accordance with our logistic regression analysis, which demonstrated that the frequency of night working was associated with shortened cycles (<25 days). Another study of workers at an optoelectronics company, also showed that rotating shift work (12 h rotating shifts) was an independent predictor of cycle irregularity (defined as MCL > 35 or < 25 days) [28].

In contrast, a recent study from Norway failed to find an association between irregular menstruations and night work, and showed no association between cycle length or menstruation duration and night work parameters. The differences may be related to different populations and shift schedules. In that study, 56 % were on a three-shift schedule, and 9 % were on a night shift, while almost all nurses experienced a three-shift schedule in the present study [25].

Our results extended previous research by conducting a follow-up investigation to detect whether there were further changes in MCL as rotating shift schedules continued to be applied for a further 2 years. Results from Part II demonstrated that in nurses in Part I whose MCL changed after starting shift rotation, had no return to the original MCL after 2 years. Only a few surveys have taken the duration of rotating shift work into account. It was mentioned above that 20+ months of rotating shift work were more likely to induce MCL changes [3], but it was found in a recent questionnaire-based survey in 43 flight attendants that irregular cycles (not exactly defined in the study) were found to occur more frequently in women whose length of work exceeding 5 years and who covered more than 14 routes per week [29].

Our results suggest the need for further investigation of how the changes in MCL are related to rotating shift work, with a view to establishing the best work patterns for the maintenance of reproductive health in nurse populations.

## Conclusion

Rotating shift work can increase the prevalence of menstrual cycle irregularity. Night shift frequency was the only risk factor associated with cycle shortening. Changes in MCL did not show recovery over a follow-up period of 2 years.

### Limitations

The main limitation of our study was the cross-sectional nature of the data. Menstrual cycle analysis was based on personal recall, which may lead to outcome misclassification. However, a previous validation study had reported that self-reports may give a better summary of MCL than menstruation diaries or calendars covering less than 2 months, and more than half of women can report their usual cycle length within 2 days of their mean cycle length [30]. Therefore, we classified the values of fluctuation into three grades defined by a 2-day cutoff. The identification of subjects for Part II was based on cycle-change characteristics from the primary investigation and the subsequent sample size was small. However, face-to-face interviews with these nurses allowed us to obtain more precise data. In addition, even though many shift nurses reported acne and hirsutism, we did not measure the level of their serum androgen to validate whether hyperandrogenism existed in shift nurses or not. Finally, the night and evening shifts were not consecutive but were interrupted with day working lasting 2 months. Under these circumstances, any changes could be alleviated, so our results may not reflect the real degree of harm accompanying rotating shift patterns. However, these rotating patterns are extensively used in Chinese hospitals after many revisions of the schedule, and may be the best for minimizing harm to the majority of nurses, while facilitating workforce management. Thus our investigation focused on the effects of intermittent evening/night shifts on menstruation under current conditions and appeared to be effective in detecting susceptible nurses.

## Availability of data and material section

The complete data supporting the conclusions of this article are not available due to involvement in unpublished research in the same field, as well as potential risk of the leaks of individual privacy.

## Additional file

Additional file 1: Questionnaire. (DOC 154 kb)

## Abbreviations

FAHSYSU: the First Affiliated Hospital of Sun Yat-sen University
GHHS: Guanghua Hospital of Stomatology
MCL: mean cycle length
BMI: body mass index
SDS: Self-rating Depression Scale
MetS: metabolic syndrome
T2DM: type 2 diabetes mellitus
ERs: emergency rooms
ICUs: intensive care units
SD: standard deviation
CI: confidence interval
ABS: absolute value
OR: odds ratio
RR: relative risk
