# Supplementary material for: Rotating shift work and menstrual characteristics in a cohort of Chinese nurses
Source: BMC Womens Health. 2016 May 4;16:24. doi: 10.1186/s12905-016-0301-y (PMC4857333; doi:10.1186/s12905-016-0301-y)
Supplement: Additional file 1: — Questionnaire. (DOC 154 kb) [file 12905_2016_301_MOESM1_ESM.doc]

Number_______________

**Preface**

**Informed consent**

This survey was conducted to investigate the satisfaction about current nursing work-schedule and its association with menstrual characteristics of female nurses, then try to give a more suitable suggestion on working schedule administration, especially about night and/or evening work, aiming to improving working conditions of nurses, as well nursing efficiency and quality.

Personal information and other content you filled in this questionnaire will be used for analysis, and be proposed to quote for publication. But information involved in personal privacy is not to be disclosed or leaked in public! You could exit the survey at any time you wanted. No compensation was offered in this study.

I am fully informed and recruited in this investigation voluntarily and solemnly.

Declarant (please sign data + job number): ___________________

(eg. 5-20-2012 AA0321)

**Questionnaire**

**Preliminary screening**

1. Are you pregnant or breastfeeding now?

A. No, Skip to the next question.

B. Yes, the survey Ended!

2. Are you continuously (more than 3 months) using hormonal drugs (like oral contraception pill, progesterone, estrogen, glucocorticoid, et al.)?

A. No, Skip to the next question.

B. Yes, Please list__________________________________________ and the survey Ended!

3. Have you already gone through menopause and/or are experiencing perimenopausal symptoms, such as hot flash, night sweat?

A. No, Please get into the **main body** of Questionnaire.

B. Yes, the survey Ended!

**Main Body**

**Part I. Basic Information**

**Q1.** Date of birth: month________ year ________

**Q2****.** Permanent residence: City________; the Year come to Guangzhou _____________

**Q3.** Ethnic group________

**A.** Han B. Hui

C. Other minority (list: ________)

**Q4.** Height ________ cm

Weight ________ kg**,** Weight fluctuation within recent 1 years________ kg (+ for gain, - for loss)

Weight before starting work________ kg

**Q5.** Highest education level________

A. Technical secondary school degree

B. Junior college degree

C. Bachelor degree

D. Master degree and above

**Q6.** Exposure to radioactive or toxic substances, such as mercury, lead?

A. No

B. Yes (Which category________, Frequency________)

C. Unclear

**Q7.** Cigarette smoking?

A. No B. Yes:

a. the Age at which you started smoking

b. Frequency______: A. 1-2 days/week B. 3-6 days/ week C. Daily

c. the number of cigarettes smoked a day_______: A. less than 10 per day B. 10－20 per day C. 20-40 per day D. more than 40 per day

**Q8.** Drinking habit________

A. Seldom or occasionally

B. Monthly

C. Weekly

D. 2-3 times per week

E. Daily

**Q9.** Regular exercise?

A. Seldom

B. 1-2 times per month

C. 3-4 times per month

D. At least 2 times per **week**

**Q10.** Sleep length: form________ o’clock to ________ o’clock

**Part II. Working Situation**

**Q11.** Ward:

A. Inpatient list name of ward_________

B. Outpatient list name of ward _________

**Q12.** Evening or night work now and/or earlier?

A. Yes. Answer in order.

B. No. Skip to **Q18.**

**Q13.** How many years of work included evening and/or night shifts during your whole life?

A. More than 10 years

B. 6-10 years

C. 3-5 years

D. 1-2 years

E. Shorter than 1 year

**Q14.** Shift schedule type_________:

A. Fixed evening shifts (from 4:00 p.m. to 12:00 a.m.)

B. Fixed night shifts (from 12:00 a.m. to 8:00 a.m.)

C. Fixed daytime shifts

D. Rotating shifts, **then answer the following question a, b and c**

**a.** Category_______: A. Two-shift rotation B. Three-shift rotation

**b.** Do there usually have one or two off days followed with a shift rotation?

A. Yes B. No (describe how_________________

**c.** Do there always have only daytime work lasting at least 1 month to alternate a 3-month schedule of rotating shifts?

A. Yes B. No (describe how_________________

**Q15.** How many evenings did you work each month on average during the past 12 months (off duty before 12:00 a.m.)?

A. No less than 7 nights

B. 6

C. 5

D. 4

E. No more than 3 nights

F. None

**Q16.** How many nights did you work each month on average during the past 12 months **(**on duty after 12:00 a.m.)?

A. No less than 7 nights

B. 6

C. 5

D. 4

E. No more than 3 nights

F. None

**Q17.** Having habit of catching up sleep after deprivation due to evening and/or night work?

A. No

B. Yes. How many hours usually: __________hours

**Q18.** Perceived job satisfaction, give a score_________（score 1-10, 1 for completely not adapted and fell exhausted; 10 for easily ）

**Q19.** Do yourself aware discomfort after work?

(If you are suffering, tick under the fittest symptoms, or leave the form blank)

| Insomnia | Dreaminess | Acne or/and hirsutism | Palpation | Fatigue | Poor appetite or dyspepsia |
| --- | --- | --- | --- | --- | --- |
|  |  |  |  |  |  |
| Irascibility | Memory loss | Shortage of breath or/and chest distress | Dizziness | Others (please list) | |
|  |  |  |  |  | |

**Part III. Current Menstruation (After starting work or under shift schedules)**

**Q20.** Age of menarche(when first got menstrual period)? _________,

the last menstrual period___________(eg. May 7^th^, 2012)

**Q21.** How many days does it usually take from the first day of your menstruation in a cycle to the first day of menstruation in the next cycle? (The onset of menstrual cycle was defined as the first of 2 consecutive days with onset of bleeding, where bleeding was more than spotting on at least one day，eg. 28 days / 28 to 30 days)

_________ days / _________ to _________ days

**Q22.** Normally, whether the number of days from the beginning of a cycle to the next cycle is as expected (identical) or not? If menstruation always occurs earlier or later than expected, what are the differences in days?

A. Regular

a. Extremely regular: no more than 1–2 days’ difference from expected

b. Very regular: maximum of 3–4 days’ difference

c. Regular: within 5–7 days

B. Irregular (changes in menstrual cycle length > 7 days)

a. Earlier than expected in most cases. maximum_________ days in advance，minimal_________ days in advance

b. Later than expected in most cases. maximum_________ days defered，minimal_________ days defered

c. It depends, earlier or later. Within_________ days

**Q23.** On average, how many days does menstrual bleeding take？(duration of the bleeding period, eg. 7 days / 5 to 7 days)

________ days / ________ to ________ days

**Q24.** On average, what is your total menstrual quantity in one cycle?

A. Less than 10 mL

B. 10–80 mL (1-6 tablespoons of menstrual fluid)

C. > 80 mL

**Q25.** Do you usually have bleeding or spotting between cycles (intermenstrual bleeding or spotting, excluding bleeding after intercourse)?

A. No, nerver or seldom

B. Yes. Then on average, how many times does it happen within half a year?

_______times

**Q26.** During menstrual bleeding, do you usually have symptoms like low back pains or/and abdominal discomforts and these symptoms last at least 2 days? How severe is it?

A. No, nerver or seldom

B. Yes

a. But no effect on normal work and life

b. Slight effect on normal work and life but no need for medicines

c. Marked effect on normal work and life or have to use of non-steroidal anti-Inflammatory drugs to alleviate pain

**Q27. Have you noticed differences in** **menstrual characteristics between before and after starting work (or within 2 years, in Part II)？**

A. No. Then skip to **Q35.**

B. Unconspicuous and temporary. Please try to describe_______________________

C. Yes. If you cross here, please answer questions in order.

**Part IV. Menstruation History** **(Before starting work or under shift schedules)**

**Q28.** How long had it taken until you noticed changes of menstrual type after beginning work (or under shift schedules)?

A. Within a month

B. 2-6 months

C. 7 months to one year

D. 13 months to two years

E. more than two years

**Q29.** After starting work (or under shift schedules), what’s the main changes of menstrual characteristics?

A. Menstrual cycle length. If you cross here, please answer **Q30**

B. Menstrual bleeding duration. Then answer **Q31**

C. Amount of flow. Then answer **Q32**

D. Intermenstrual bleeding or spotting. Then answer **Q33**

E. Dysmenorrhea intensity. Then answer **Q34**

**Q30.**

**I.** Prior to working (or under shift schedules), how many days did it take from the first day of your menstruation in a cycle to the first day of menstruation in the next cycle normally?

________ days / ________ to ________ days

**II.** Prior to working (or under shift schedules), whether the number of days from the beginning of a cycle to the next cycle was as expected (identical) or not on average? If menstruation occurred earlier or later than expected, what was the difference in days?

A. Regular.

a. Extremely regular: no more than 1–2 days’ difference from expected

b. Very regular: maximum of 3–4 days’ difference

c. Regular: within 5–7 days

Then skip to **Q30 III**.

B. Irregular (changes in menstrual cycle length > 7 days).

a. Earlier than expected in most cases. maximum ________days in advance，minimal ________ days in advance

b. Later than expected in most cases. maximum ________ days defered，minimal _____days defered

c. It depends, earlier or later. Within________ days

**Q30** finished, please skip to **Q35** if there has been no other change of menstrual type since after starting work (or under shift schedules). If other changes has presented, please back to **Q29** and follow instructions to fill in the blank.

**III**. Since starting work (or under shift schedules), has menstrual cycle changed to be shorter or longer on average? How many days has shortened or prolonged?

A. Shorter. ________ days

B. Longer. ________ days

**Q30** finished, please skip to **Q35** if there has been no other change of menstrual type since after starting work (or under shift schedules). If other changes has presented, please back to **Q29** and follow instructions to fill in the blank.

**Q31.** Since starting work (or under shift schedules) , has menstrual bleeding days changed to be shorter or longer on average? How many days has shortened or prolonged?

A. Shorter. ________ days

B. Longer. ________ days

**Q31** finished, please skip to **Q35** if there has been no other change of menstrual type since after starting work (or under shift schedules). If other changes has presented, please back to **Q29** and follow instructions to fill in the blank.

**Q32.** Since starting work (or under shift schedules), has the amount of flow changed to be less or more?

A. Less

B. More

**Q32** finished, please skip to **Q35** if there has been no other change of menstrual type since after starting work (or under shift schedules). If other changes has presented, please back to **Q29** and follow instructions to fill in the blank.

**Q33.** After starting work (or under shift schedules), how many times does bleeding or spotting between cycles happen within half a year on average?

________ times

**Q33** finished, please skip to **Q35** if there has been no other change of menstrual type since after starting work (or under shift schedules). If other changes has presented, please back to **Q29** and follow instructions to fill in the blank.

**Q34.** After starting work (or under shift schedules), does symptoms of low back pains or/and abdominal discomforts alleviate or aggravate?

A. Alleviate

B. Aggravate

**Q34** Finished, please skip to **Q35** if there has been no other changes of menstrual type since after starting work (or under shift schedules). If other changes has presented, please back to **Q29** and follow instructions to fill in the blank.

**Q35.** Does changed menstruation tend to recover?

A. No

B. Yes

**Part V. Reproductive History**

**Q36.** Have ever had sexual life before?

A. Yes. Answer in order.

B. No. Skip to **Part VI.**

**Q37. M**arriage?

A. Yes, marriage age __________

B. No

**Q38.** Constant contraception?

A. No. Skip to **Q39**

B. Yes, Type: _________(eg. condom, IUD, ligation or taking oral contraception pills). Skip to **Q40**

**Q39.** Failure in pregnancy even with regular sexual life without contraception

A. Yes, How long________

B. No

**Q40.** Times of pregnancy ________ times

A. Zero. Skip to **Part VI Q42.**

B. At least one time. List and then answer in order.

First pregnancy: month________ year________

Last pregnancy: month________ year________

**Q41.**

I. Number of birth (≥28 gestational weeks)_________

| Live Birth or not | Gestational weeks | Cesarean section | Vaginal delivery |
| --- | --- | --- | --- |
| Eg. Yes | 37^+3^ | √ |  |
| 1. |  |  |  |
| 2. |  |  |  |

II. Number of Abortion (before 28 gestational weeks)__________

| Gestational weeks | Spontaneous Abortion | Artificial abortion operation | Medical termination | Ectopic pregnancy |
| --- | --- | --- | --- | --- |
|  |  |  |  |  |
|  |  |  |  |  |
|  |  |  |  |  |

**Part VI. Diagnosed Diseases and Operation History**

**Q42.** Filled in form according to hints if you are having/ever had the following diseases, or leave the form blank

| **Obstetrical & Gynecological**  **Diseases** | | | **When Diagnosed** | **Conservative therapy (if still taking medicines, list)** | **Surgical therapy (operation name)** | **Cured or not, When** |
| --- | --- | --- | --- | --- | --- | --- |
| Polycystic Ovarian Syndrome (PCOS) | | |  |  |  |  |
| Progesterone deficiency | | |  |  |  |  |
| Hyperprolactinemia | | |  |  |  |  |
| Ovarian tumor | Benign | | Type: |  | Procedure:  A. Conventional (open)  B.Laparoscopy  Site: __________ |  |
|  | Malignant | | Type: |  |  |  |
| Premature Ovarian Failure (POF) | | |  |  |  |  |
| Endometriosis (EMT) | | |  |  |  |  |
| Fallopian Tubes | | |  |  | Procedure:  A. Conventional (open)  B.Laparoscopy  Site: __________ |  |
| Adenomyosis | | |  |  |  |  |
| Asherman’s syndrome | | |  |  |  |  |
| Endometrial hyperplasia | | |  |  | A. Hysteroscopy operation  B. D&G  C.___________ |  |
| Endometrial carcinoma | | | Type: |  |  |  |
| Uterine myoma | | Intramural |  |  | A. Myomectomy  B. _________**__** |  |
|  |  | Subserous |  |  |  |  |
|  |  | Submucous |  |  |  |  |
| Cervical intraepithelial neoplasia III | | |  |  |  |  |
| Cervical cancer | | | Type: |  |  |  |
| Trichomonal vaginitis | | |  |  |  |  |

**Q43.** Filled in form according to hints if you are having/ever had the following diseases, or leave the form blank

| **Other system Disorders** | **Official Diagnosis** | **When Diagnosed** | **Cured or not, When** | **Conservative or surgical therapy** |
| --- | --- | --- | --- | --- |
| Pituitary Gland |  |  |  |  |
| Thyroid |  |  |  |  |
| Adrenal Gland |  |  |  |  |
| Breast |  |  |  |  |
| Blood system or clotting disorders |  |  |  |  |
| Cardiovascular diseases ( eg, Hypertension) |  |  |  |  |
| Digestive system （especially, hepatic disorders） |  |  |  |  |
| Diabetes Milltus |  |  |  |  |
| Others, please give a description |  | | | |

**Part VII. Family History**

**Q44.** Having family members who suffer from menstrual disorders?

A. No

B. Yes. Symptoms _________

Relationship __________

**Q45.** Do there have diagnosed hereditary diseases in your family?

A. No

B. Yes. Name of diseases________________

**Part VIII. Self-Rating Depression Scale (SDS)**

**Q46.** Please tick the options the best fit for you

|  | **Never or Seldom** | **Sometimes**  **(****1-2 days/week)** | **Usually**  **(3-4 days/week)** | **Always**  **(5-7 days/week)** | **Score** |
| --- | --- | --- | --- | --- | --- |
| 1. Depressed mood |  |  |  |  |  |
| 2. Morning symptoms |  |  |  |  |  |
| 3. Crying |  |  |  |  |  |
| 4. Insomnia |  |  |  |  |  |
| 5. Diminished Appetite |  |  |  |  |  |
| 6. Sexual interest |  |  |  |  |  |
| 7. Weight loss |  |  |  |  |  |
| 8. Constipation |  |  |  |  |  |
| 9. Palpitations |  |  |  |  |  |
| 10. Fatigue |  |  |  |  |  |
| 11. Clouded reasoning |  |  |  |  |  |
| 12. Difficulty with completing tasks |  |  |  |  |  |
| 13. Restlessness |  |  |  |  |  |
| 14. Lack of hope |  |  |  |  |  |
| 15. Irritability |  |  |  |  |  |
| 16. Difficult decision making |  |  |  |  |  |
| 17. Diminished self esteem |  |  |  |  |  |
| 18. Life satisfaction |  |  |  |  |  |
| 19. Suicidal ideation |  |  |  |  |  |
| 20. Anhedonia |  |  |  |  |  |

**The End! Thanks for you cooperation!**

**Investigator Sign _____________**

**Date _____________**
